# Effect of pterygium on corneal astigmatism, irregularity and higher-order aberrations: a comparative study with normal fellow eyes

**DOI:** 10.1038/s41598-023-34466-4

**Published:** 2023-05-05

**Authors:** Chang Ho Yoon, Bo Ram Seol, Hyuk Jin Choi

**Affiliations:** 1 Department of Ophthalmology, Seoul National University College of Medicine, Seoul, South Korea; 2 Laboratory of Ocular Regenerative Medicine and Immunology, Seoul Artificial Eye Center, Seoul National University Hospital Biomedical Research Institute, Seoul, South Korea; 3 Department of Ophthalmology, VHS (Veterans Health Service) Medical Center, Seoul, South Korea; 4 Department of Ophthalmology, Seoul National University Hospital Healthcare System Gangnam Center, 39th Fl., Gangnam Finance Center, 152 Teheran-ro, Gangnam-gu, Seoul, 06236 South Korea

**Keywords:** Conjunctival diseases, Corneal diseases

## Abstract

Pterygium is an abnormal growth of fibrous conjunctival tissue that invades the cornea, resulting in corneal distortion, astigmatism, and increased higher-order aberrations (HOAs). However, few studies have compared eyes with pterygium to normal fellow eyes when interpreting HOAs and there is no study that revealed the effect of the thickness or grading of the pterygium on the change of HOAs. Therefore, we evaluated the effects of nasal pterygium by comparing the normal fellow eye of 59 patients. The pterygium significantly increased with-the-rule corneal astigmatism and corneal irregularity. Trefoils, horizontal coma, and quatrefoils were significantly induced by the pterygium. The grading of the pterygium was not correlated with its characteristics except for the thickness. In multiple linear regression analysis, pterygium-induced corneal astigmatic/irregularity values and horizontal trefoil/quatrefoil were associated with the area of the pterygium. The length of the pterygium was an independent inducer of oblique trefoil/quatrefoil, while horizontal coma was independently associated with both its length and width. The thickness was not correlated with any optical parameters. Together, the results demonstrate that nasal pterygium significantly induces corneal astigmatism, irregularity and some HOAs. These pterygium-associated changes in optical parameters could be predicted by the length, width and area of the pterygium.

## Introduction

Pterygium is characterized by abnormal fibrous conjunctival tissue that invades from the limbus to the central cornea^1^. The anterior corneal surface is the most powerful image-forming interface in the eye and it is the primary determinate of the quality of the retinal image^2^. When pterygium invasion crosses the pupillary margin, visual acuity is definitely decreased by blocking the visual axis. Although pterygium does not involve the visual axis, visual quality is also decreased when pterygium induces corneal distortion, corneal astigmatism and/or increased higher-order aberrations (HOAs)^3^.

The wavefront aberration is the distance in micrometers between the actual wavefront and the ideal wavefront^4^. In contrast to low-order aberrations such as astigmatism, which is easily correctable with spectacles, HOAs are not correctable with spectacles. Previous studies have reported that pterygium induces HOAs, especially coma and trefoil^5–7^. The amount of corneal astigmatism and ocular wavefront aberrations have shown a significant correlation with the size of the pterygium^5,7,8^. As the cornea is symmetric between the right and left eyes^9^, parameter changes caused by pterygium may be more clearly analyzed when eyes with pterygium are compared with normal contralateral eyes. However, few studies have compared eyes with pterygium to normal fellow eyes when interpreting HOAs induced by pterygium^5,10–12^. Moreover, only one study evaluated differences in HOAs between eyes and the effect of the size and length of the pterygium on HOAs in subjects with unilateral pterygium, ^12^ and there has been no study that revealed the effect of the thickness or grading of the pterygium on the change of HOAs.

Accordingly, we aimed to investigate the effect of pterygium on astigmatism, irregularity, and HOAs related to its length, width, area, and thickness by comparing it with the data from contralateral normal eyes.

## Results

### Demographic data

Fifty-nine eyes with nasal pterygium (51 primary and 8 recurrent pterygia) and 59 normal fellow eyes of 59 subjects were enrolled in this study. Table 1 shows the demographics, characteristics of the pterygium, and the topographical parameters of the patients. The mean (± SD) age was 62.1 ± 9.2 years (range, 37–81 years). There were 27 men and 32 women. The mean (± SD) length, width, area, and thickness of the pterygium were 2.93 ± 1.08 mm, 4.56 ± 1.38 mm, 9.58 ± 4.80 mm^2^, and 0.41 ± 0.15 mm, respectively.

**Table 1:** Demographics, characteristics of pterygium, and comparisons of corneal astigmatism and irregularities between eyes.

	Normal contralateral eyes (n = 59)	Pterygium eyes (n = 59)	*p*
Age (years)	62.1 ± 9.2		
Male:Female	27:32		
Pterygium			
Length (mm)		2.93 ± 1.08	
Width (mm)		4.56 ± 1.38	
Area (mm^2^)		9.58 ± 4.80	
Thickness (mm)		0.41 ± 0.15	
Corneal astigmatism^a^			
With-the-rule (n)	23	42	
Oblique (n)	9	13	< 0.001*
Against-the-rule (n)	27	4	
Magnitude (D)	0.84 ± 0.47	2.07 ± 1.33	< 0.001^†^
J0	0.02 ± 0.42	0.76 ± 0.72	< 0.001^††^
J45	− 0.02 ± 0.25	− 0.32 ± 0.57	< 0.001^†^
Irregularity of the 3 mm zone (D)^b^	1.53 ± 0.61	3.67 ± 2.26	< 0.001^†^
Irregularity of the 5 mm zone (D)^b^	1.87 ± 0.68	5.04 ± 2.72	< 0.001^†^

D, diopter.

^a^Measured by the ATLAS 9000 Corneal Topography System.

^b^Measured by the Orbscan II Slit Scanning Corneal Topography/Pachymetry System.

*McNemar-Bowker test.

^†^Wilcoxon signed-rank tests.

^††^Paired t-test.

### Differences in corneal astigmatism, irregularity, and wavefront aberration

Compared to the normal fellow eye, pterygium significantly induced with-the-rule (WTR) or oblique corneal astigmatisms (*p* < 0.001, McNemar-Bowker test). Magnitudes of the corneal astigmatism were significantly higher in the eyes with pterygium (2.07 ± 1.33 D) than in the normal fellow eyes (0.84 ± 0.47 D). J0 and J45 corneal astigmatisms in eyes with pterygium (0.76 ± 0.72 and -0.32 ± 0.57 D) were significantly higher than those in normal fellow eyes (0.02 ± 0.42 and -0.02 ± 0.25 D) (all *p* < 0.001). Irregularities of the 3- and 5-mm zones were also significantly higher in eyes with pterygium (3.67 ± 2.26 and 5.04 ± 2.72 D vs. 1.53 ± 0.61 and 1.87 ± 0.68 D, all *p* < 0.001) (Table 1).

**Figure 1:**
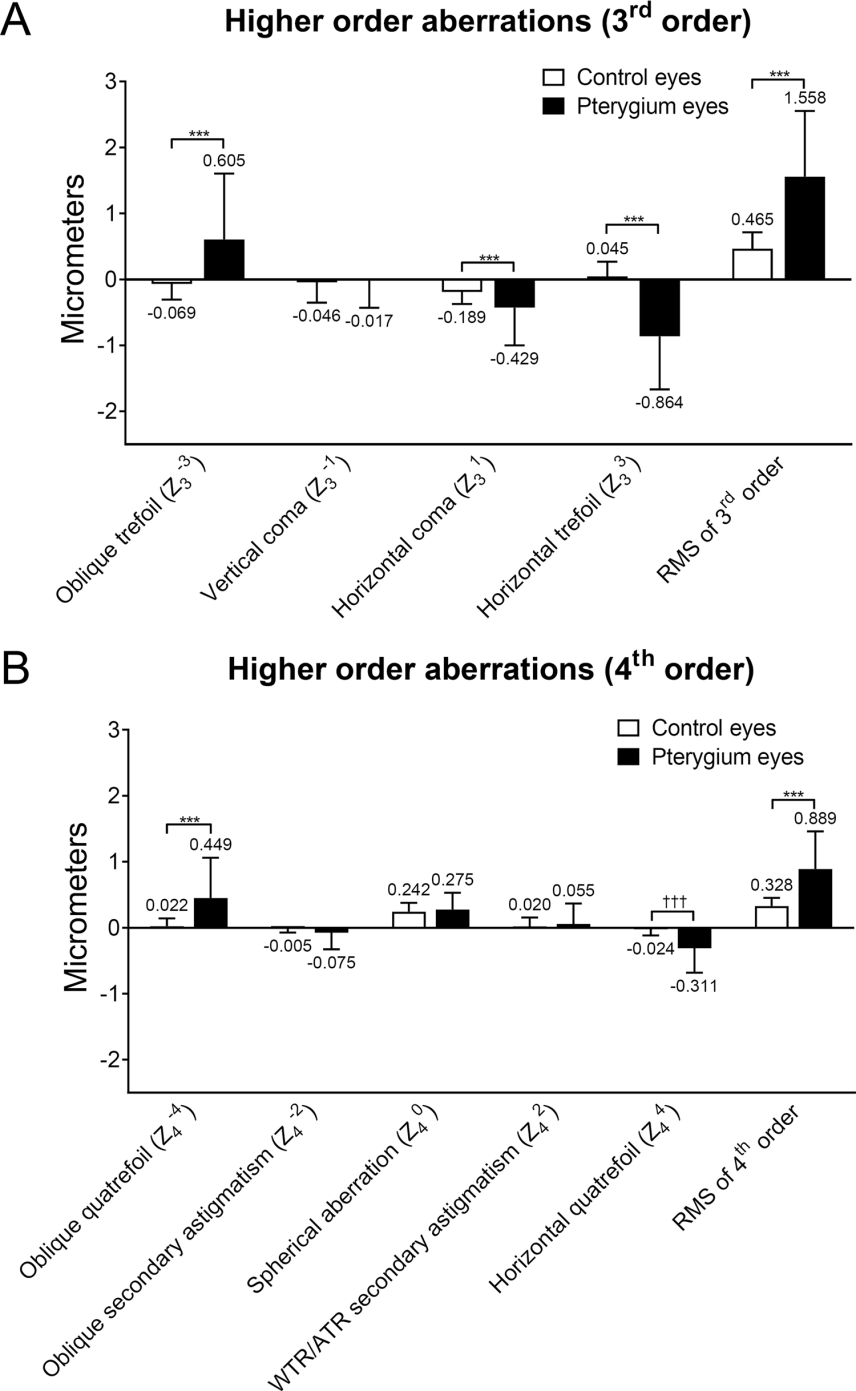
Differences in corneal higher order aberrations between eyes with pterygium and normal fellow eyes. (**A**) Third order aberration. (**B**) Fourth order aberration. RMS, root mean square; WTR, with-the-rule; ATR, against-the-rule. Wilcoxon signed-rank test (***p < 0.001) or Paired t-test (^†††^*p* < 0.001) was used. To compare the right and left eyes, enantiomorphism of higher-order aberrations was neutralized.

**Figure 2:**
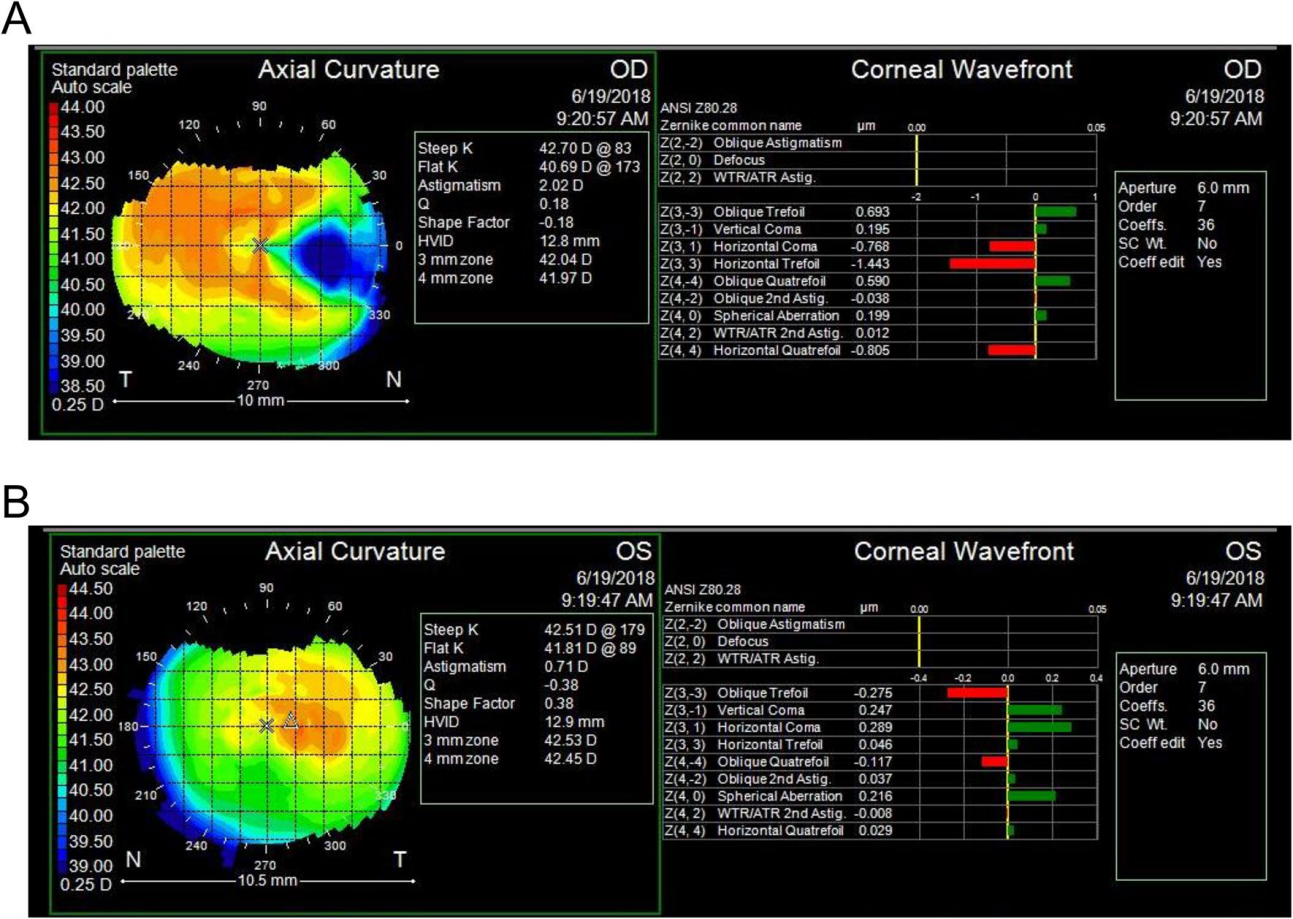
Representative case showing the effect of pterygium on corneal higher-order aberrations. (**A**) Right eye with pterygium. (**B**) Normal left eye. Compared with the normal fellow eye, pterygium induced significant with-the-rule astigmatism. Moreover, the right eye showed much higher values of oblique trefoil and oblique quatrefoil and much lower values of horizontal coma, horizontal trefoil and horizontal quatrefoil than the normal left eye after enantiomorphism correction of the left eye. Vertical coma and spherical aberrations were not much different between the eyes.

Regarding HOAs, compared to the values of the HOAs in the normal fellow eyes, those with oblique and horizontal trefoils, horizontal coma, root mean square (RMS) of the 3rd order aberrations, oblique and horizontal quatrefoils, and RMS of the 4th order aberrations were significantly higher in eyes with pterygium (all *p* < 0.05) (Fig. 1). Vertical coma, spherical aberration, oblique secondary astigmatism, and WTR/against-the-rule (ATR) secondary astigmatism were not different between the two eyes. A representative case is shown in Fig. 2.

There were no differences in the corneal astigmatic values, irregularity values or the HOAs between eyes with primary and recurrent pterygium (data not shown, all *p* > 0.05, Mann–Whitney U test).

### Effect of pterygium on induced corneal astigmatism, irregularity and wavefront aberration

With regard to the grading of the pterygium, it was T1 in 20 patients, T2 in 31 patients, and T3 in 8 patients. The length, width, and area of the pterygium were not different according to the grading of the pterygium. Only the thickness of the pterygium was significantly different among the groups (*p* < 0.001, Kruskal–Wallis test). In Dunn’s post hoc test, the T1 group (0.300 ± 0.089 mm) was significantly thinner than in the T2 (0.480 ± 0.131 mm) and T3 (0.465 ± 0.173 mm) groups (*p* < 0.001 and *p* = 0.042, respectively); however, the T2 and T3 groups were not different (*p* > 0.999). The grading of the pterygium was not associated with any of the astigmatic, irregularity or HOA values, which were significantly induced by pterygium (Table 2). Considering the close correlation between the thickness and grading of the pterygium, only the thickness was used for the later analyses, as described below.

**Table 2:** Effect of the pterygium grading on the induced corneal optical parameters.

	T1 (n = 20)	T2 (n = 31)	T3 (n = 8)	*p*
Length (mm)	3.195 ± 1.246	2.809 ± 1.166	3.182 ± 1.199	0.478*
Width (mm)	10.806 ± 4.974	8.247 ± 4.393	12.285 ± 7.421	0.478*
Area (mm^2^)	4.764 ± 1.226	4.356 ± 1.497	4.862 ± 1.256	0.156*
Thickness (mm)	**0.300 ± 0.089**	**0.480 ± 0.131**	**0.465 ± 0.173**	** < 0.001**^†^
Δ Magnitude of corneal astigmatism (D)	1.153 ± 1.189	1.187 ± 1.402	1.564 ± 1.878	0.969^†^
Δ J0 corneal astigmatism (D)	0.832 ± 0.566	0.582 ± 0.701	1.109 ± 0.865	0.055^†^
Δ J45 corneal astigmatism (D)	− 0.372 ± 0.548	− 0.222 ± 0.581	− 0.477 ± 0.482	0.932^†^
Δ Irregularity of 3 mm zone (D)	2.240 ± 2.434	2.132 ± 2.367	1.900 ± 1.594	0.542^†^
Δ Irregularity of 5 mm zone (D)	3.605 ± 2.545	2.958 ± 2.852	2.875 ± 1.483	0.204^†^
Δ Oblique trefoil (μm)	0.909 ± 0.700	0.442 ± 1.041	0.956 ± 1.493	0.050^†^
Δ Horizontal coma (μm)	− 0.018 ± 0.578	− 0.381 ± 0.618	− 0.268 ± 0.415	0.139^†^
Δ Horizontal trefoil (μm)	− 0.986 ± 0.727	− 0.770 ± 0.829	− 1.238 ± 1.071	0.375^†^
Δ RMS of the 3rd order aberrations (μm)	1.085 ± 0.745	0.991 ± 1.050	1.493 ± 1.363	0.465^†^
Δ Oblique quatrefoil (μm)	0.504 ± 0.399	0.342 ± 0.639	0.557 ± 1.077	0.232^†^
Δ Horizontal quatrefoil (μm)	− 0.349 ± 0.311	− 0.209 ± 0.433	− 0.426 ± 0.427	0.273*
Δ RMS of the 4th order aberrations (μm)	0.571 ± 0.387	0.470 ± 0.515	0.875 ± 0.922	0.225^†^

D, diopter; RMS, root mean square.

Δ: value of the pterygium eye-value of the normal fellow eye.

*One way ANOVA.

^†^Kruskal–Wallis test.

Bold means significant *p* values < 0.05.

In the univariate linear regression analysis, the length, width, and area of the pterygium were significantly correlated with all induced astigmatic and irregularity parameters (Δ magnitude of corneal astigmatism, J0/J45 corneal astigmatisms and corneal irregularities of the 3- and 5-mm zones) (Table 3). The length, width, and area of the pterygium were also correlated with some HOAs induced by pterygium. In contrast, the thickness was not significantly correlated with any induced corneal optical parameters.

**Table 3:** Effect of pterygium on induced ocular optical parameters.

	Univariate linear regression								Multivariate linear regression							
	Length (mm)		Width (mm)		Area (mm^2^)		Thickness (mm)		Length (mm)		Width (mm)		Area (mm^2^)		Thickness (mm)	
	β	*p*	β	*p*	β	*p*	β	*p*	β	*p*	β	*p*	β	*p*	β	*p*
Δ Magnitude of the corneal astigmatism (D)	**0.510**	** < 0.001**	**0.360**	**0.006**	**0.514**	** < 0.001**	0.016	0.908	(–)		(–)		**0.514**	** < 0.001**	(–)	
Δ J0 corneal astigmatism (D)	**0.519**	** < 0.001**	**0.564**	** < 0.001**	**0.574**	** < 0.001**	− 0.073	0.603	(–)		**0.320**	**0.034**	**0.359**	**0.018**	(–)	
Δ J45 corneal astigmatism (D)	− **0.465**	** < 0.001**	− **0.277**	**0.035**	− **0.420**	**0.001**	0.082	0.559	− **0.465**	** < 0.001**	(–)		(–)		(–)	
Δ Irregularity of the 3 mm zone (D)	**0.637**	** < 0.001**	**0.464**	** < 0.001**	**0.656**	** < 0.001**	− 0.174	0.208	(–)		(–)		**0.656**	** < 0.001**	(–)	
Δ Irregularity of the 5 mm zone (D)	**0.462**	** < 0.001**	**0.384**	**0.003**	**0.490**	** < 0.001**	− 0.165	0.233	(–)		(–)		**0.490**	** < 0.001**	(–)	
Δ Oblique trefoil (μm)	**0.421**	**0.001**	0.152	0.254	**0.405**	**0.002**	− 0.123	0.380	**0.421**	**0.001**	(–)		(–)		(–)	
Δ Horizontal coma (μm)	**0.343**	**0.008**	− 0.203	0.127	0.192	0.150	− 0.261	0.059	**0.598**	** < 0.001**	− **0.505**	** < 0.001**	(–)		(–)	
Δ Horizontal trefoil (μm)	− **0.493**	** < 0.001**	− **0.485**	** < 0.001**	− **0.564**	** < 0.001**	0.019	0.890	(–)		(–)		− **0.564**	** < 0.001**	(–)	
Δ RMS of the 3rd order aberrations (μm)	**0.605**	** < 0.001**	**0.518**	** < 0.001**	**0.669**	** < 0.001**	− 0.049	0.729	(–)		(–)		**0.669**	** < 0.001**	(–)	
Δ Oblique quatrefoil (μm)	**0.306**	**0.020**	0.067	0.618	0.240	0.069	− 0.035	0.805	**0.306**	**0.020**	(–)		(–)		(–)	
Δ Horizontal quatrefoil (μm)	− **0.288**	**0.028**	− **0.313**	**0.017**	− **0.360**	**0.005**	0.030	0.829	(–)		(–)		− **0.360**	**0.005**	(–)	
Δ RMS of the 4th order aberrations (μm)	**0.682**	** < 0.001**	**0.397**	**0.002**	**0.651**	** < 0.001**	− 0.111	0.428	**0.682**	** < 0.001**	(–)		(–)		(–)	

NA, not applied; D, diopter; RMS, root mean square.

Δ: value of the pterygium eye-value of the normal fellow eye.

Bold means significant *p* values < 0.05.

In the multivariate linear regression analysis, the area of the pterygium was independently associated with the change of all astigmatic and irregularity values except Δ J45 corneal astigmatism of which the length was the only inducer. Δ J0 corneal astigmatism was also significantly increased by the width. Regarding HOAs, only the length of the pterygium was an independent inducer of oblique trefoil and quatrefoil, while only the area of the pterygium was significantly associated with pterygium-induced horizontal trefoil and quatrefoil. Δ Horizontal coma was significantly affected by both the length and width of the pterygium. In addition, the area and length independently induced RMSs of the 3rd and 4th order aberrations, respectively. Interpreting the results using the RMS of the 3rd-order aberrations as an example, it means the area of the pterygium was the only independent predictor of the change in the RMS of the 3rd-order aberrations, and the difference was estimated as follows: the difference in the RMS of the 3rd-order aberrations (μm) = − 0.085 + 0.124 × area of the pterygium (mm^2^).

## Discussion

Considering that the pterygium primarily involves the corneal surface, in this study, we focused on pterygium-induced changes in corneal optical parameters compared with normal fellow eyes. Pterygium significantly increased the magnitude of corneal astigmatism and induced J0/J45 corneal astigmatism. Irregularities of the 3- and 5-mm zones were also significantly increased by pterygium. Moreover, pterygium significantly induced some 3rd- and 4th-order HOAs (trefoils, horizontal coma, and quatrefoils), which may affect the quality of vision. The area of the pterygium was an independent contributing factor to changes in most of astigmatic and irregularity parameters, horizontal trefoil/quatrefoil, and RMS of the 3rd order aberrations, while the length of the pterygium was independently related to an increase in oblique trefoil/quatrefoil and RMS of the 4th order aberrations. Δ Horizontal coma was significantly induced by both the length and width of the pterygium. The thickness of the pterygium was not associated with any induced corneal optical parameters.

It has been well documented that the corneal topographic data and HOAs of the anterior cornea, especially for 3rd- and 4th-order terms, of right and left eyes have mirror-image symmetry^13^. Therefore, in this study, we designated normal fellow eyes of the same patients as controls to more exactly investigate the effect of the pterygium itself on the corneal optical parameters. Considering that there is tremendous variability in eye aberrations from person to person, HOAs significantly change with age, and surgery itself would induce unexpected aberrations, the present study might suggest more reasonable data than the results described by other studies in which the pterygium-induced changes were estimated by the effect of pterygium excision^7^ or in comparisons with age- and sex-matched controls^8^.

Several studies have established a relationship between the size of the pterygium and corneal astigmatism^14,15^. Mohammad-Salih and Sharif^14^ reported that the total area, extension, and width of the pterygium showed a strong correlation with the magnitude of corneal astigmatism measured by keratometry. They suggested that the total area and length have a stronger correlation with corneal astigmatism than width, which is similar to our findings. In the present study, the magnitude and J0 corneal astigmatism were increased in eyes with pterygium, which indicates that nasal pterygium induces WTR astigmatism. On the other hand, J45 corneal astigmatism was decreased in eyes with pterygium, which means that nasal pterygium in the right eye leads to increased counterclockwise oblique astigmatism^16^.

It has also been well documented that corneal irregularity and distortion caused by pterygium induce HOAs, which may be responsible for the deterioration in the quality of the vision^6,7,10^. In the same manner, this study demonstrated that pterygium significantly increases corneal irregularities of the 3- and 5-mm zones and thus induces some HOAs. Considering that all pterygia included in this study were located in the nasal quadrant, resulting in asymmetric corneal distortion, we could expect that pterygia mainly affect corneal wavefront aberrations that are not symmetric across the eye. As expected, we found that absolute values of most of horizontally asymmetric aberrations (horizontal coma, horizontal trefoil, and oblique quatrefoil) were significantly higher in eyes with pterygium, while some horizontally symmetric aberrations, including vertical coma and spherical aberrations, were not affected by pterygium. Regarding spherical aberration, pterygium had no effect, as reported elsewhere^5,6^. This implies that surgeons may choose the asphericity of the intraocular lens that reduces postoperative ocular spherical aberrations according to the current topographic data, regardless of whether cataract surgery is performed simultaneously with excision of the pterygium or scheduled alone in cases with a small pterygium. In this study, pterygium-induced 3rd-order HOAs were greater than the 4th-order HOAs, and the first two HOAs showing the greatest change in absolute value were horizontal and oblique trefoil, which are consistent with the results reported in previous studies^5,7^.

Theories around the causes of the corneal distortion and flattening induced by pterygium include the tractional force of contractile elements, the localized pooling of tears at the pterygium apex, and stromal scarring^14,17,18^. Interestingly, in the present study, pterygium-induced changes in corneal optical parameters were mostly associated with the length and area of the pterygium, whereas the thickness and grading of the pterygium was not related to any induced corneal optical parameters. This implies the morphology of the head rather than the body or tail of the pterygium may be the key to determining aforementioned factors affecting the corneal distortion and flattening. The facts that pterygia usually exhibit firm adhesion to the anterior corneal stroma, while spanning the limbal region without adherence^18^, pterygium with a flat corneal scleral transition zone induced more corneal scarring and astigmatism than pterygium with a nodular appearance^18^, and traction by body or tail evoked by temporal gaze is not an important factor in the change of astigmatism, ^19^ may support the importance of the head morphology. Meanwhile, the thickness of the pterygium was closely linked to the grading of the pterygium in this study, which has been known to be predictive of recurrence after pterygium excision. ^20^. Therefore, we think above mentioned negative result also has clinical significance. That is, the thickness of the pterygium itself may have clinical significance as one of predictive parameters for the recurrence after pterygium excision.

As HOAs have been known to be associated with glare, halos, and other various visual symptoms, pterygium-induced HOAs can also affect the visual quality of the patient. In particular, total RMS and coma have been known to be responsible for night vision disturbances^21^. Horizontal coma, but not vertical coma, has been reported to be associated with double vision in patients who undergo refractive surgery^22^. In the present study, we found that horizontal coma was significantly induced by pterygium, while vertical coma was not different between eyes with pterygium and normal fellow eyes. Moreover, RMSs of the 3rd and 4th order, trefoils, and quatrefoils were also significantly increased in eyes with pterygium, which would explain the cause of the visual symptoms in patients with pterygium who suffer from visual disturbance and low visual quality despite glasses correction.

With regard to determining the optimal time of pterygium excision, information about HOAs may provide surgeons with a valuable tool. In fact, Pesudovs and Figueiredo^7^ suggested that surgeons consider the removal of pterygia before they grow to 4 mm in size to avoid residual aberrations. Considering that patients with significant subjective visual complaints after corneal refractive surgery had an increase in RMS of the 3rd-order aberrations of 0.63 μm compared to those without symptoms, ^23^ surgical excision of the pterygium would be expected to improve the visual function when the area of the pterygium is greater than 5.76 mm^2^ according to the regression formula described above. This might provide additional valuable insight to surgeons during their decision-making process.

There are some limitations of this study. First, the measurement of HOAs for 6 mm diameter with a Placido disc-based topography is improbable to be accurate especially when the pterygium is large. These may be fundamental limitations of all studies which deal with the effect of the pterygium on astigmatism or corneal/ocular HOAs using a Placido disc-based topography^5,7,8,19,24^. However, we think corneal wavefront data in this study could be interpretable and have clinical significance as a subgroup analysis (the small versus large pterygium group according to the involvement of central 6 mm zone) showed almost the same results between two groups, even allowing for exaggeration in the eye with large pterygium (Supplementary Fig. 1). Second, slit lamp photography-based measurement of the size of the pterygium might not be accurate, considering that Fuchs flecks and the pterygium cap may not be evident on slit lamp photographs and the position of the limbus under the pterygium is judged by projecting the limbal position from where it is located above and below the pterygium. In line with this, in vivo confocal microscopy (IVCM) may be a valuable tool for accurately measuring the size of the pterygium as it has been well known that IVCM identifies Fuchs flecks sensitively and measurements of the pterygium are generally larger in confocal images than in anterior segment photographs^25,26^. Third, we could not evaluate whole ocular aberrations because the aberrometer (iDesign aberrometer; Johnson & Johnson Vision Care, Inc., Santa Ana, CA) in our clinic repeatedly showed acquisition errors in most patients, especially those with large pterygia. Therefore, the direct effect of pterygium-induced corneal HOAs on the whole ocular optical system could not be investigated. Last, we only included patients with unilateral nasal pterygium. Lesions located at other sites may show different results from those of this study.

In conclusion, through a comparative study with normal fellow eyes, pterygium had a significant effect on corneal astigmatism, irregularity and HOAs. Most of the differences in astigmatism, irregularity and HOAs are related to the length or area of the pterygium, while the thickness of the pterygium has no effect. These findings may be helpful in explaining the visual symptoms in patients with pterygium and for deciding about the timing of the surgical excision of the pterygium.

## Methods

### Study population

This retrospective case–control study was conducted at the Department of Ophthalmology, Seoul National University Hospital. This study adhered to the tenets of the Declaration of Helsinki, and approval for a retrospective review of clinical records was obtained from the Seoul National University Hospital Institutional Review Board (IRB No. 1212–070–451). Owing to the retrospective design of the study and the use of deidentified patient information, the Seoul National University Hospital Institutional Review Board waived the need for written informed consent.

Patients with unilateral nasal pterygium and normal fellow eyes, who visited our institute from 2011 to 2020, were included. Eligible patients needed to have imaging of both eyes using the Orbscan II Slit Scanning Corneal Topography/Pachymetry System (Orbscan, Inc., Salt Lake City, UT, USA) and the ATLAS 9000 Corneal Topography System (Carl Zeiss Meditec, Inc., Dublin, California, USA), as well as Visante anterior segment optical coherence tomography (Carl Zeiss Meditec) imaging and anterior segment photography using the SL-D7 slit lamp (Topcon, Tokyo, Japan) in the eye with pterygium.

The exclusion criteria included (1) a history of corneal refractive or other ocular surface surgery except primary pterygium removal; (2) temporal or bilateral pterygium; (3) corneal ectasia and scarring; and (4) other corneal and ocular surface diseases that may have affected the study parameters.

### Measurements of the pterygium size and thickness

**Figure 3:**
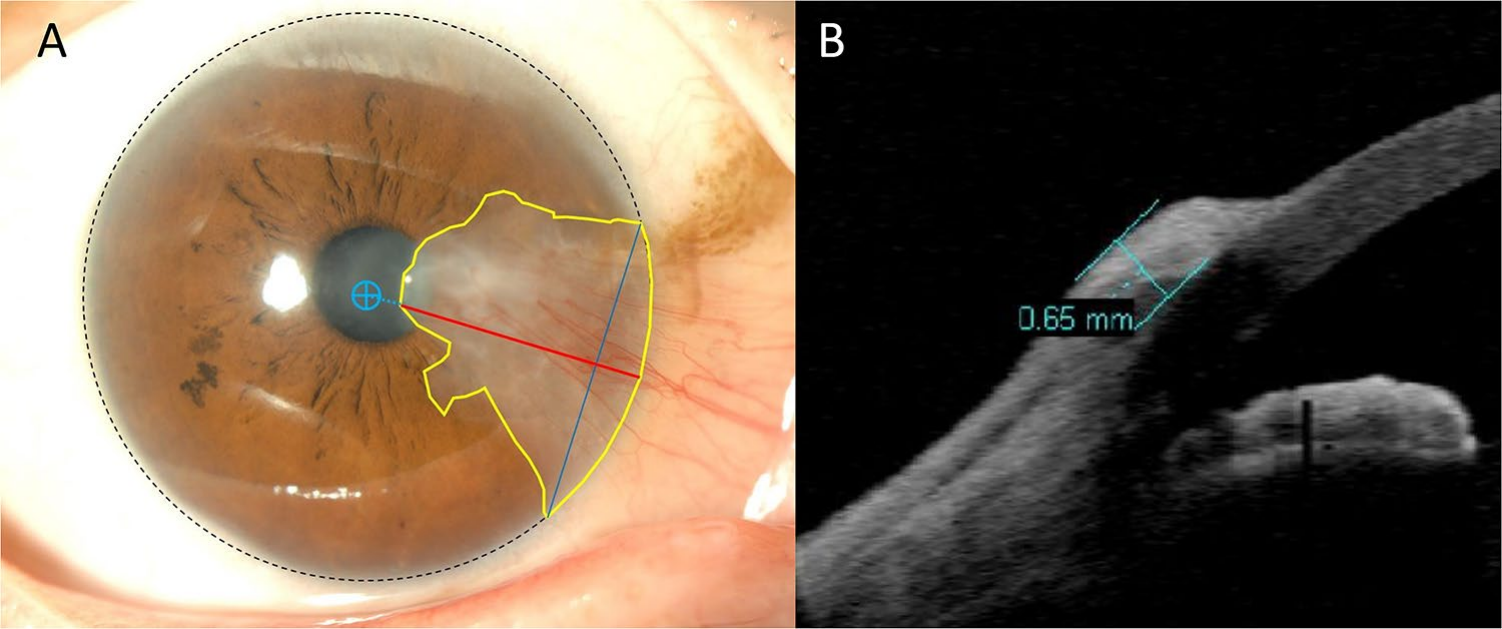
Anterior segment photography and anterior segment optical coherence tomography of an eye with pterygium. (**A**) The length (the maximum pterygium’s length, *red line*, from the leading edge of the pterygium and the farthest point of the limbus), width (*blue line*, between the opposing edges of the pterygium intersecting the limbus), and area (*yellow line*) of the pterygium were calculated. Blue target, geographical center of the cornea. (**B**) The thickness was measured at the thickest point of the pterygium.

Anterior segment images were taken with an SL-D7 slit lamp at the primary position. During photography, a fixed target was used to standardize the primary position. The length, width, and area of the pterygium were measured using NIH ImageJ software (ImageJ, National Institutes of Health, Bethesda, Maryland, USA), as described elsewhere with a little modification^10^. Briefly, the maximum pterygium’s length (i.e. the length) was defined as the distance from the leading edge of the pterygium and the farthest point of the limbus. The width was defined as the distance between the opposing edges of the pterygium intersecting the limbus. The area, which was defined as the surface area of the cornea involved by the pterygium, was calculated using the freehand tool (Fig. 3A). The values were used by averaging the measurements performed by two independent investigators (CHY and HJC). Discrepancies between reviewers were resolved by consensus or adjudication by the third investigator (BRS) if the difference between the two values was greater than 10% of the mean.

The thickness of the pterygium was defined as the distance from the conjunctival surface to the outline of the outer corneosclera at the thickest point and measured using Visante anterior segment optical coherence tomography (Fig. 3B).

### Grading of the pterygium

The pterygium was categorized according to the grading system described by Tan et al^20^. The grading system categorized the pterygium as follows based on the relative translucency of the tissue: Grade T1 (episcleral vessels underlying the pterygium body were clearly distinguished and unobscured); Grade T2 (episcleral vessels underlying the pterygium body were indistinctly seen or partially obscured); and Grade T3 (episcleral vessels underlying the pterygium body were totally obscured). The preoperative photographs were independently reviewed by 2 investigators (CHY and HJC). Discrepancies between reviewers were resolved by consensus or adjudication by the third investigator (BRS).

### Measurements and analyses of corneal optical parameters

Corneal astigmatism was defined by the presence of WTR (60°–120°), oblique (30°–60° or 120°–150°), or ATR (0°–30° or 150°–180°) keratometric data of the ATLAS 9000 Corneal Topography System. Power vector analysis was used to compare astigmatism as described by Thibos et al^27^. In this analysis, a Jackson cross-cylinder oriented at 180 degrees (referred to as J0), which quantifies WTR and ATR astigmatism, and a Jackson cross-cylinder oriented at 45 degrees (referred to as J45), which quantifies oblique astigmatism, were used.

Corneal irregularities at the 3- and 5-mm zones were measured using the Orbscan II Slit Scanning Corneal Topography/Pachymetry System.

Corneal wavefront errors in the 6 mm optical zone were also measured using an ATLAS 9000 Corneal Topography System. Corneal wavefront aberrations were decomposed into Zernike polynomials to the fourth order, and each HOA value was compared between the two eyes. Analyzed HOAs were oblique trefoil (Z_3_^–3^), vertical coma (Z_3_^–1^), horizontal coma (Z_3_^1^), horizontal trefoil (Z_3_^3^), RMS of the 3rd order aberrations, oblique quatrefoil (Z_4_^–4^), oblique secondary astigmatism (Z_4_^–2^), spherical aberration (Z_4_^0^), WTR/ATR secondary astigmatism (Z_4_^2^), horizontal quatrefoil (Z_4_^4^), and RMS of the 4th order aberrations. Regarding repeatability of measurements of HOAs, repeated measurements were performed in 14 eyes with pterygia and the intraclass correlation coefficient (ICC) showed 0.949 or more (all *p* < 0.001; excellent reliability) for all HOAs except for horizontal quatrefoil (ICC of 0.83, *p* < 0.001; good reliability)^28,29^.

To compare the right and left eyes, we neutralized the enantiomorphism of the corneal astigmatism and the HOA. The left eye data were transformed by mirroring the vectors on the y-axis to avoid cancellation due to the sign when averaging the results^30^.

### Statistical analysis

The McNemar-Bowker test was used to compare the distribution of the astigmatism types between the eyes with pterygium and the contralateral eyes. The normality of numerical data distribution was checked with the Shapiro–Wilk test. A paired t-test or Wilcoxon signed-rank test was used to compare the continuous variables (astigmatic, irregularity, and wavefront values) between the two eyes. A Mann–Whitney U test was conducted to evaluate the corneal astigmatic values, irregularity values or the HOAs differences between eyes with primary and recurrent pterygium. Regarding the parameters that showed significant differences between the two eyes, the difference values (Δ) were calculated by subtracting each value of the normal fellow eye from that of the eye with pterygium and they were used for further analyses.

One-way ANOVA with Tukey's multiple comparison post hoc tests or Kruskal–Wallis test with Dunn's post hoc test was used to compare continuous variables according to the grading of the pterygium. Univariate and multivariate linear regression analyses were carried out to analyze the independent contributing factors associated with the pterygium-induced changes in each corneal optical parameter. Parameters with a *p* value < 0.2 in the univariate analysis were included in a multiple regression analysis by stepwise selection. Statistical significance was accepted at *p* < 0.05. The data are presented as the mean ± standard deviation (SD). GraphPad Software (GraphPad Prism, Inc., La Jolla, CA, USA) was used for statistical analyses.

## Supplementary Information

Supplementary Information.

## Data Availability

The data that support the findings of this study are available from the corresponding author upon reasonable request.
