# Supplementary material for: Effect of pterygium on corneal astigmatism, irregularity and higher-order aberrations: a comparative study with normal fellow eyes
Source: Sci Rep. 2023 May 5;13:7328. doi: 10.1038/s41598-023-34466-4 (PMC10163024; doi:10.1038/s41598-023-34466-4)
Supplement: Supplementary file 1 — Supplementary Information. [file 41598_2023_34466_MOESM1_ESM.pdf]

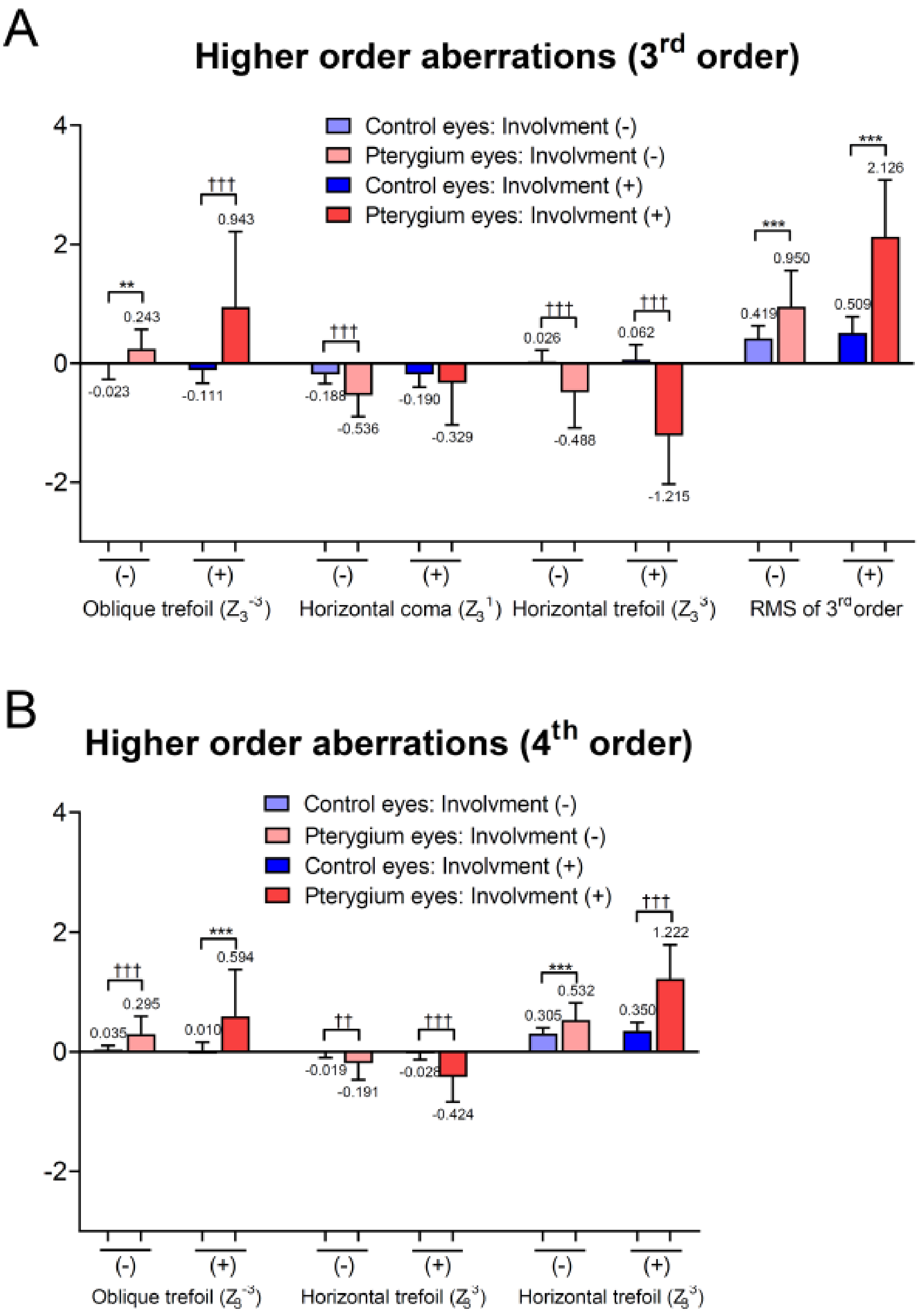

Supplementary Figure 1. Subgroup analysis according to the involvement of central 6 mm zone.

(A) Third order aberration. (B) Fourth order aberration.

L, the length of the pterygium; RMS, root mean square

Wilcoxon signed-rank test (***p*<0.01, ****p*<0.001) or Paired t-test (††*p*<0.01, †††*p*<0.001) was used.

To compare the right and left eyes, enantiomorphism of higher-order aberrations was neutralized.
